# Spatiotemporal distribution, trend, forecast, and influencing factors of transboundary and local air pollutants in Nagasaki Prefecture, Japan

**DOI:** 10.1038/s41598-023-27936-2

**Published:** 2023-01-16

**Authors:** Santos Daniel Chicas, Jair Gaspar Valladarez, Kiyoshi Omine, Venkataraman Sivasankar, Sangyeob Kim

**Affiliations:** 1 IRI-THESys and Geography Department, Humboldt-Universität zu Berlin, Unter den Linden 6, 10099 Berlin, Germany; 2 Faculty of Science and Technology, University of Belize, Belmopan, Belize; 3 Department of Civil Engineering, School of Engineering, Nagasaki University, 1-14 Bunkyo, Nagasaki, 852-8521 Japan; 4 Post Graduate and Research Department of Chemistry, Pachaiyappa’s College (affiliated to University of Madras), Chennai, Tamil Nadu 600030 India; 5 Estuary Research Center, Shimane University, Nishikawatsu-cho 1060, Matsue, 690-8504 Japan

**Keywords:** Environmental sciences, Environmental social sciences

## Abstract

The study of PM_2.5_ and NO_2_ has been emphasized in recent years due to their adverse effects on public health. To better understand these pollutants, many studies have researched the spatiotemporal distribution, trend, forecast, or influencing factors of these pollutants. However, rarely studies have combined these to generate a more holistic understanding that can be used to assess air pollution and implement more effective strategies. In this study, we analyze the spatiotemporal distribution, trend, forecast, and factors influencing PM_2.5_ and NO_2_ in Nagasaki Prefecture by using ordinary kriging, pearson's correlation, random forest, mann–kendall, auto-regressive integrated moving average and error trend and seasonal models. The results indicated that PM_2.5_, due to its long-range transport properties, has a more substantial spatiotemporal variation and affects larger areas in comparison to NO_2_, which is a local pollutant. Despite tri-national efforts, local regulations and legislation have been effective in reducing NO_2_ concentration but less effective in reducing PM_2.5_. This multi-method approach provides a holistic understanding of PM_2.5_ and NO_2_ pollution in Nagasaki prefecture, which can aid in implementing more effective pollution management strategies. It can also be implemented in other regions where studies have only focused on one of the aspects of air pollution and where a holistic understanding of air pollution is lacking.

## Introduction

Studies have attributed the increase in air pollution to rapid urban development and modernization^1^. Over the years, much emphasis has been placed on the analysis of PM_2.5_ (particulate matter with a diameter of 2.5 μm or less) and NO_2_ (Nitrogen Oxide) due to the adverse effects on public health^2–4^, global climate^5,6^ and long-range transport, particularly for PM_2.5_^7^. As a result of public health and environmental implications, countries and international organizations have engaged in regulating and monitoring PM_2.5_ and NO_2_ concentrations. For instance, the World Health Organization (WHO) air quality guidelines established in 2005 were revised and released on September 22, 2021. These new guidelines come from decades of research showing that air pollution's health effects result from high exposure and very low concentrations^8^. Therefore, the guidelines of 2005 recommended that the annual average of PM_2.5_ and NO_2_ concentrations should not exceed 10 and 40 μg/m^3^ (21 ppb), respectively. The 2021 guidelines reduce these recommendations to 5 and 10 μg/m^3^ (5 ppb) for PM_2.5_ and NO_2_, respectively.

The analysis and monitoring of PM_2.5_ and NO_2_ are essential to assess the effectiveness of mitigation strategies and compliance with standards. Currently, monitoring stations reliably and accurately measure PM_2.5_ and NO_2_ concentrations. However, monitoring is often difficult as PM_2.5_ and NO_2_ measurements are only done at some locations due to the high costs of installation, maintenance, and management of monitoring stations. As a result, detailed information about the spatiotemporal distribution, trend, and climatic and temporal effect of PM_2.5_ and NO_2_ is often lacking in locations with few monitoring stations. Therefore, the need exists, in these locations, to implement a multi-method approach that could predict PM_2.5_ and NO_2_ where it is not measured and generate fine-grain spatial distribution data, examining the changes of PM_2.5_ and NO_2_ concentration over time and understand the climatic and temporal effects on PM_2.5_ and NO_2_ concentrations. This information is critical for identifying areas (hotspots) that do not comply with international pollution concertation standards, quantitatively evaluating the air quality policy, and assessing the risks to human health^9^. Furthermore, empirical models are required to describe the general features of the spatial patterns of PM_2.5_ and NO_2_, trends, and influencing factors^10,11^.

In Japan and many regions of the world, studies have only focused on the spatiotemporal distribution, trend, forecast, or influencing factors of PM_2.5_ and NO_2_^12,13^. However, rarely studies have combined these to generate a more holistic understanding that can help identify health risk areas, influencing factors, pollution trends, and the efficacy (or lack thereof) of policy interventions^11^. For instance, in Nagasaki Prefecture, most of the studies have only focused on either the health impact caused by air pollutants^14–16^, the long-range transport of air pollutants from the Asian continent^17–19^, or the effects of climatic variables and the spatial and temporal distribution of PM_2.5_^20^. Although these studies provide essential information, a multi-method approach is necessary to understand better the distribution, factors, and current and future trends of PM_2.5_ and NO_2_ pollution in Nagasaki Prefecture.

This study used a multi-method approach to analyze PM_2.5_ and NO_2_ data in Nagasaki Prefecture from 2013 to 2021. This study aims (1) to estimate PM_2.5_ and NO_2_ pollution variability in unmeasured areas using ordinary kriging, (2) to identify and analyze the correlation of the major climatic and temporal factors that influence PM_2.5_ and NO_2_ pollution in Nagasaki Prefecture via Pearson's correlation and random forest feature selection and (3) to conduct a trend and forecast analysis of PM_2.5_ and NO_2_ based on fitting loess, automated auto-regressive integrated moving average (ARIMA) and error trend and seasonal models (ETS). Using a multi-method approach, we provide a broader analysis and understanding of the spatiotemporal distribution, forecast, trend, and influencing factors of pollutants which is crucial for the improvement, development, and assessment of mitigation strategies and for identifying health risk areas. Furthermore, this proposed multi-method approach can be used in other regions where studies have only focused on one of the aspects of air pollution and where a holistic understanding of air pollution is lacking.

### Policy background

**Figure 1:**
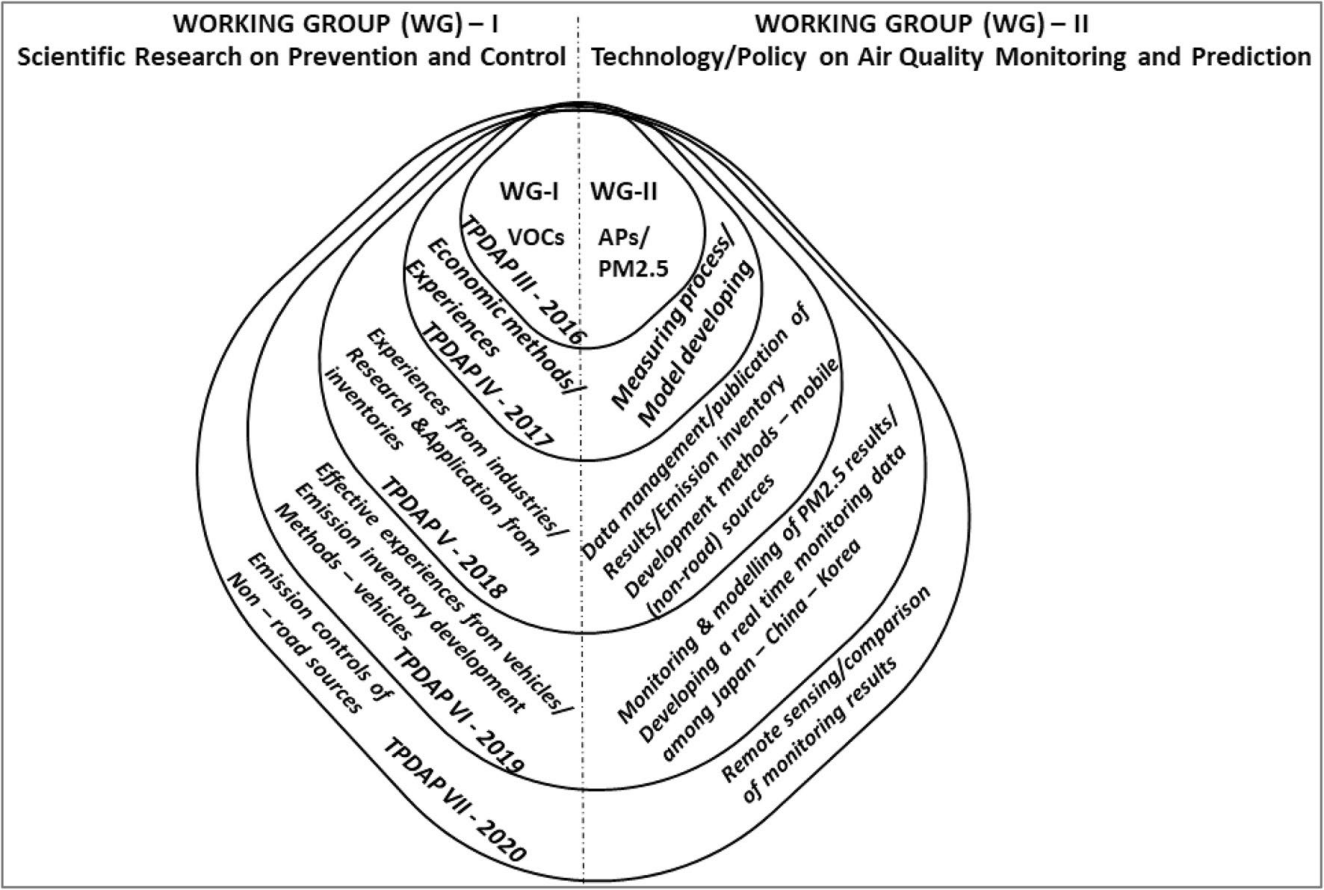
Tripartite Policy Dialogue on Air Pollution working groups one and two.

As a result of the rapid economic development of the Northeast Asian sub-region and the resulting environmental problems, China, Japan and Korea have held a Tripartite Environment Ministers Meeting (TEMM) annually since 1999^21^. This meeting aims to strengthen environmental cooperation among these countries and address environmental problems at the domestic, regional, and global levels. At the 15th Tripartite Environment Ministers Meeting in 2013, the Tripartite Policy Dialogue on Air Pollution (TPDAP) was established and started in 2014^21^. The objective of the establishment of the TPDAP was to coordinate efforts among the three countries to address the air pollution problem by developing cooperation initiatives and sharing information about air pollution policy implementation and impacts. The 3rd TPDAP in 2016 established two working groups to share air pollution information (Fig. 1).

The establishment of the Tripartite Policy Dialogue on Air Pollution (TPDAP) has resulted in reducing air pollution in the three countries (Table 1).

**Table 1:** Significant policies implemented by China, Japan, and Korea to improve air quality.

Country	Policy	Targeted sources	Impact	Research
Japan	Since the introduction of the "Comprehensive Policy Effort on PM_2.5_" in 2013, policies and measures were introduced that fall under three categories: -Domestic measures -International cooperation -Reinforcing scientific knowledge	-Stationary -Vehicles -Vessels -Open burning -NH_3_ -Regional	Since 2013, the annual average of PM_2.5_ concentration has decreased, with the number of occasions issuing public alerts on PM_2.5_ decreasing from 37 in 2013 to 2 in 2017	Declining PM_2.5_ as a result of policy implementation: 2010–2018 declining PM_2.5_^22^ 2003–2018 declining PM_2.5_^23^
China	Since the introduction of the "Action Plan on the Prevention and Control of Air Pollution" in 2013, significant policies and measures have been introduced which fall under five categories: -Rule of law -Scientific and Technological support -Comprehensive emission reduction -Innovative management -Social participation	-Industry -Energy sector -Vehicles -Non-point source pollution	The average concentration of PM_2.5_ in 74 cities decreased by 42% from 2013 to 2018. These cities are applying Ambient Air Quality Standard	Declining PM_2.5_ 2013–2018^24^ Declining PM_2.5_ 2019–2020^25^
Korea	Since the revision of the "Air Quality Preservation Act" in 2013 and the introduction of "Comprehensive Plan on Fine Dust Management Implementation" in 2017, strategic policies and measures have been implemented which fall under four categories: -Reduction of domestic emissions -Public health -International cooperation -Solid foundation and communication	-Industry -Transport -Power generation -Agriculture and daily surrounding -Indoor air quality -Regional	PM_2.5_ reduction from 2010 to 2019 has shown minimal changes each year, with the PM_2.5_ level decreasing by 2 μg/m^3^ in 2018 from the level of 2017. This is due to the implementation of the comprehensive plan in 2017	PM_2.5_ decrease 2003–2017^26^ PM_2.5_ decrease 2010–2020^27^

## Results

### PM_2.5_ and NO_2_ spatiotemporal distribution

The years that had the maximum mean average level of PM_2.5_ concentration were 2014 and 2016, with 16.2 and 14 μg/m^3^, respectively. The minimum mean average concentrations in 2020 and 2021 were 10.4 and 9.7 μg/m^3^, respectively (Table 2). There have been dynamic temporal variations of PM_2.5_ concentration with respect to a yearly minimum, maximum, and mean during the study period. These variations are also expressed in the year's seasons, with Winter and Spring being the seasons with the highest PM_2.5_ concentrations. For each year of the study period, 2013–2021, Spring had the highest concentration of PM_2.5_ (Table S2). Amidst these variations, there is an indication of a declining trend of PM_2.5_ concentration from 2013 to 2021.

**Table 2:** Annual descriptive statistics for PM_2.5_ and NO_2_.

	PM_2.5_			NO_2_		
Year	Minimum	Maximum	Mean	Minimum	Maximum	Mean
2013	6	24	12.6	2	26	6.3
2014	11	24	16.2	1	21	6.3
2015	8	19	13.7	2	21	6.1
2016	11	21	14	2	20	5.8
2017	10	19	12.9	2	20	6
2018	7	19	11.8	2	19	5.6
2019	8	18	11.8	0	18	5.8
2020	8	14	10.4	1	17	4.7
2021	7	14	9.7	1	15	4.4

**Figure 2:**
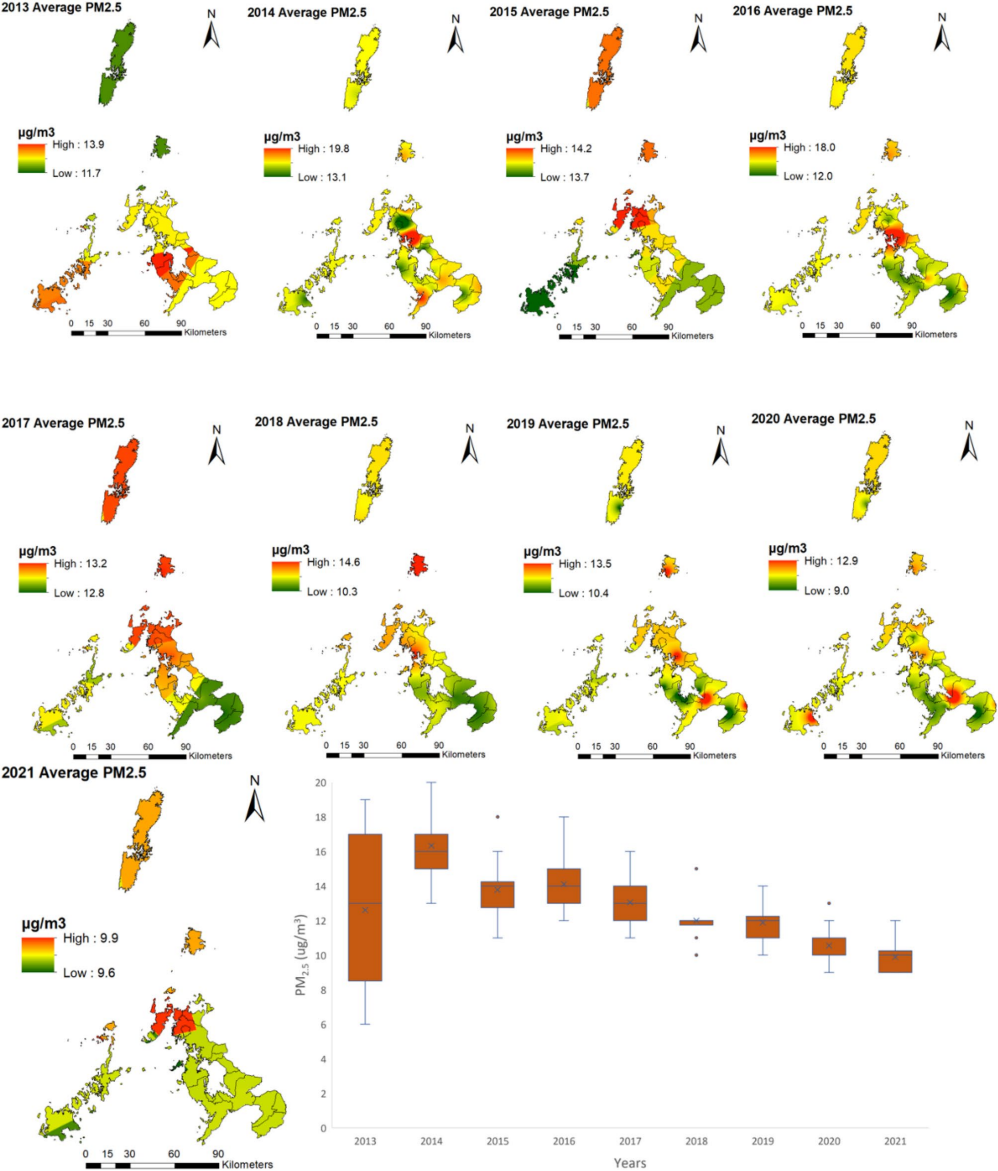
Spatiotemporal distribution of PM_2.5_ from 2013 to 2021 in Nagasaki Prefecture, Japan. Created with ArcMap 10.7 (https://www.arcgis.com/index.html).

Regarding the spatial distribution of PM_2.5_ concentration, there is also yearly variation as to the hotspot of PM_2.5_ (Fig. 2). However, to a great degree, the most affected area is the westernmost part of Nagasaki Prefecture, as illustrated by the PM_2.5_ spatial distribution maps of 2015, 2016, 2017, and 2018.

**Figure 3:**
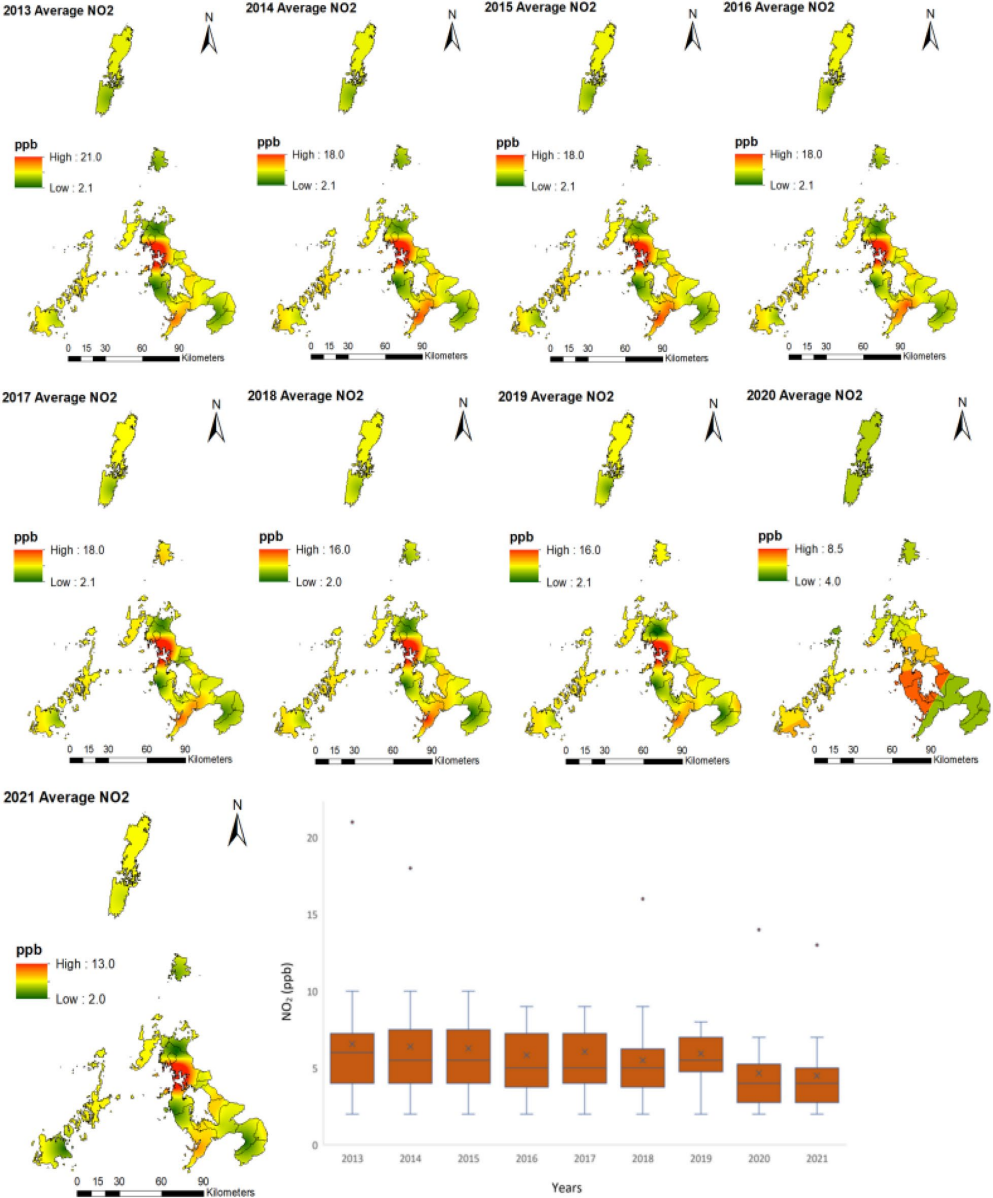
Spatiotemporal distribution of NO_2_ from 2013 to 2021 in Nagasaki Prefecture, Japan. Created with ArcMap 10.7 (https://www.arcgis.com/index.html).

Concerning NO_2_, the maximum mean average level of NO_2_ concentrations was in 2013 and 2014 at 6.3 ppb. The minimum mean average concentrations were in 2020 and 2021, with 4.7 and 4.4 ppb, respectively (Table 2). The box plots indicated minimal temporal variations of NO_2_ concentration during the study period (Fig. 3). Winter and Spring are the seasons with the highest NO_2_ concentration, with Winter being the season with the highest NO_2_ concentrations for each year, except for 2013 when Spring had the highest concentration (Table S2). The spatial distribution of NO_2_ indicated that the hotspots have remained in the same location over the years (Fig. 3). The high concentration of NO_2_ is located in Sasebo and Nagasaki, the two largest cities of Nagasaki Prefecture.

### Pearson's correlation and Random forest

**Figure 4:**
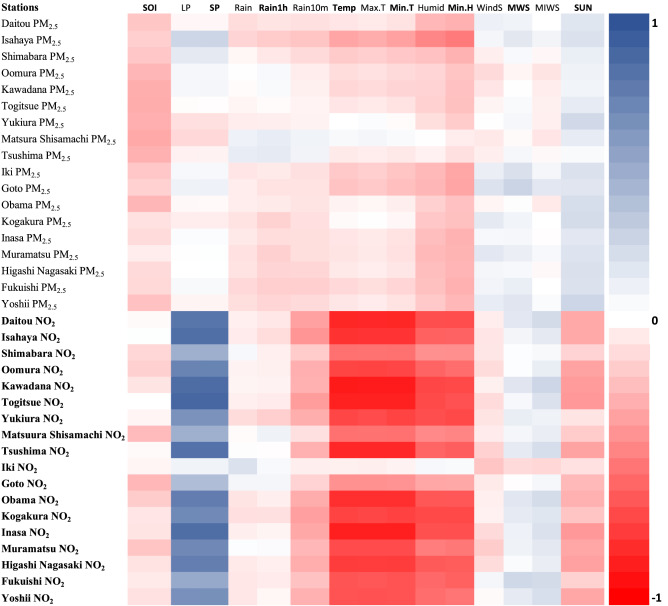
Heatmap represents the correlation between climatic factors and PM_2.5_ and NO_2_ air pollution data for 18 monitoring stations in Nagasaki Prefecture, Japan. * SOI = Souther Oscillation Index, LP = Average local pressure, SP = Average sea level pressure, Rain = Daily Precipitation, Rain1h = Maximum 1-h precipitation, Rain10m = Maximum 10 min precipitation, Temp = Average Temperature, Max.T = Maximum Temperature, Min.T = Minimum Temperature, Humid = Average humidity, Min.H = Minimum humidity, WindS = Average wind Speed, MWS = Minimum wind speed, MIWS = Maximum instantaneous wind speed, SUN = Sunlight time.

Pearson's correlation results indicated that meteorological factors influence PM_2.5_ and NO_2_ concentrations in Nagasaki Prefecture (Fig. 4). In the case of PM_2.5_, the factors had weak positive, negative, and mixed correlation results. The factors negatively correlated with PM_2.5_ were the southern oscillation index, average temperature, maximum temperature, minimum temperature, average humidity, and minimum humidity, with the southern oscillation index having the most substantial influence among these factors. On the other hand, minimum wind speed and sunlight time had a positive correlation. The other factors had mixed results in some stations having positive, negative, and no correlation. For NO_2_, some factors had a strong positive and negative correlation, with others having a weak positive and negative correlation. Average temperature, maximum temperature, minimum temperature, average humidity, and minimum humidity had a strong negative correlation, with average local pressure and average sea level pressure having a strong correlation. On the other hand, the southern oscillation index, rain maximum 10 min, average wind speed, and sunlight time had a weak negative correlation, with maximum wind speed and maximum instantaneous wind speed having a weak positive correlation. The other factors had mixed results, with some stations having positive, negative, or no correlation.

**Figure 5:**
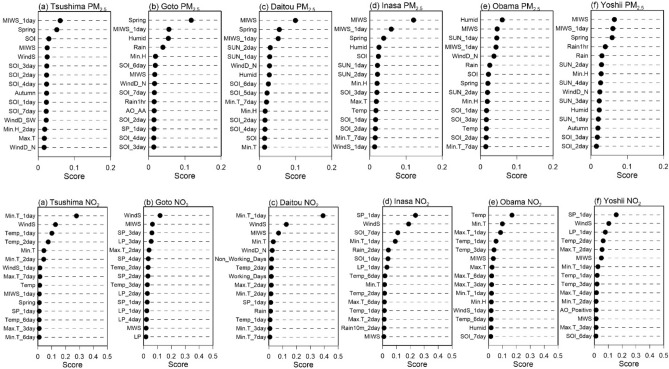
RF feature selection for PM2.5 and NO_2_ of different monitoring stations in Nagasaki Prefecture, Japan.

The RF model feature selection results at Tsushima, Goto, Daitou, Inasa, Obama, and Yoshii for PM_2.5_ and NO_2_ are shown in (Fig. 5). These stations were selected based on their location, which are representative of the study area. Among the monitoring stations, the most important predicting factors for PM_2.5_ were Spring, maximin instantaneous wind speed one day, maximum instantaneous wind speed, humidity, sunlight time one day, and southern oscillation index. At the observation tower of Tsushima, Goto, Inasa, and Yoshii, Spring is among the three major predicting factors of PM_2.5,_ with Spring being the primary predictor at Goto. Maximum instantaneous wind speed was among the three most important predictors for Goto, Daitou, Insas, and Yoshii and was the primary predictor at Tsushima. Maximum instantaneous wind speed was the main predictor for Daitou, Inasa, and Yoshii and the second most important predictor for Obama. Humidity was the main predictor of Obama and the third predictor at Goto. Southern oscillation index was only a significant predictor at Tsushima.

On the other hand, for NO_2,_ the three most important predictors that were among the stations were average wind speed, minimum temperature one day, maximum instantaneous wind speed, average sea level pressure one day, average temperature one day, average sea level pressure three days, southern oscillation index seven days, average temperature, maximum temperature one day and average local pressure one day. Average wind speed was among the three main predictors in Tsushima, Goto, Daitou, Inasa, and Yoshii, and the main predictor in Goto. Minimum temperature was the major predictor in Tsushima and Daitou. In Goto and Daitou, maximum instantaneous wind speed was the second and third major predictor, respectively. Sea level pressure one day was the main predictor in Inasa and Yoshii. Average temperature one day, average sea level pressure three days, southern oscillation index seven days, average temperature, maximum temperature one day, and average local pressure one day were among the three most important factors in only one of the stations. The feature selection result from RF indicated that the factors influencing PM_2.5_ and NO_2_ concentrations in Nagasaki Prefecture vary depending on the location of the monitoring stations.

Tables 3 and 4 show the results of the random forest models for each of the 18 stations for PM_2.5_ and NO_2_, respectively. Model accuracy was evaluated using R^2^ and MSE. In the case of PM_2.5_, the result indicated that the accuracy estimates for the 18 stations are varied with R^2^ values in the range of 0.41–0.53 and MSE of 22.7–37.6 for the training dataset and R^2^ values ranging from 0.16 to 0.33 and MSE 32.7–51.3 for the test dataset. The low values of R^2^, high values for MSE, and the high difference of R^2^ between the trained model and the test model indicated that the RF models constructed with these factors could not be used to predict PM_2.5_ concentrations.

**Table 3:** Result of the random forest model for PM_2.5_ using hyperparameters of the optimum value.

Point (PM_2.5_)	Train R^2^	Test R^2^	Train MSE	Test MSE
Daitou	0.53	0.33	33.2	51.3
Isahaya	0.49	0.29	29.6	43.1
Shimabara	0.49	0.31	29.4	40.1
Oomura	0.50	0.28	24.5	32.7
Kawadana	0.50	0.27	23.6	33.0
Togitsue	0.49	0.22	23.5	35.9
Yukiura	0.47	0.18	24.0	35.1
Matsuura Shisamachi	0.47	0.25	28.8	37.1
Tsushima	0.43	0.16	35.2	61.5
Iki	0.43	0.18	37.6	61.2
Goto	0.41	0.16	32.7	51.1
Obama	0.47	0.27	22.7	29.4
Kogakura	0.47	0.24	29.7	39.2
Inasa	0.46	0.21	30.3	43.4
Muramatsu	0.48	0.23	24.6	36.1
Higashi Nagasaki	0.48	0.24	25.5	36.3
Fukuishi (Jihai)	0.47	0.23	36.0	53.1
Yoshii	0.46	0.23	26.7	32.1

**Table 4:** Result of the random forest model for NO_2_ using hyperparameters of the optimum value.

Point (NO_2_)	Train R^2^	Test R^2^	Train MSE	Test MSE
Daitou	0.709	0.564	4.865	7.979
Isahaya	0.803	0.735	2.458	3.393
Shimabara	0.725	0.542	1.411	2.181
Oomura	0.717	0.608	2.348	3.772
Kawadana	0.745	0.58	1.124	1.678
Togitsue	0.807	0.63	1.753	3.451
Yukiura	0.541	0.359	0.38	0.511
Matsuura Shisamachi	0.586	0.423	0.969	1.524
Tsushima	0.711	0.587	1.362	2.066
Iki	0.671	0.495	2.284	3.255
Goto	0.602	0.357	3.341	3.577
Obama	0.656	0.524	0.403	0.674
Kogakura	0.665	0.499	4.913	7.814
Inasa	0.51	0.354	9.396	8.8
Muramatsu	0.525	0.43	4.121	2.95
Higashi Nagasaki	0.735	0.654	3.156	4.635
Fukuishi(Jihai)	0.673	0.617	9.277	10.43
Yoshii	0.669	0.566	0.95	1.35

Whereas, in the case of NO_2_, R^2^ was higher (Test: 0.354–0.735) than the R^2^ values of PM_2.5_; thus, the factors used in this study are a better predictor of NO_2_ concentration than PM_2.5_. However, the R^2^ values for most of the NO_2_ test models are still low to be used to predict NO_2_ concentrations, except for the results of Isahaya, which can be considered acceptable.

### Trend and Forecast analysis

At a 0.05 significance level, the Mann–Kendall test determined that PM_2.5_ and NO_2_ in most of the monitoring stations had a monotonic trend and a negative slope (Table 5). The stations with no monotonic trend for PM_2.5_ were Shimabara, Oomura, Kawadana, Togitsue, MatsuuraShimachi, and Tsushima, and for NO_2_ were Yukiura, Tsushima, Iki, Obama, and Muramatsu. The stations that had the most significant magnitude of reduction for PM_2.5_ were Daitou (− 1.278 μg/m^3^), Fukuishi Jihai (− 1.178 μg/m^3^), and Kogakura (− 1.01 μg/m^3^), while Goto (− 0.43 μg/m^3^) had the lowest. For NO_2_, the most significant magnitude of reduction was observed in Fukuishi Jihai (− 0.78 ppb), Higashi Nagasaki (− 0.50 ppb), and Kogakura (− 0.49 ppb), and the lowest in Matsuura Shisamachi (− 0.12 ppb). Figures S1 and S2 represent the data decomposition and tend for six monitoring stations.

**Table 5:** PM_2.5_ and NO_2_ Sen slope, Mann Kendal and Forecast of the monitoring stations in Nagasaki Prefecture, Japan.

Location	Seasonal Sen slope	Correlated seasonal Mann-Kendal	MAPE trend STL	ADF test	Models	MAPE	RMSE
Daitou_PM_2.5_	− 1.278	0.001	0.1321	0.01	ETS(M,A,M)	15.1101	1.8093
Isahaya_PM_2.5_	− 0.697	0.012	0.1820	0.01	ARIMA(1,1,1)(2,0,0)[12]	16.6838	2.2761
Shimabara_PM_2.5_	− 0.626	0.151	0.1413	0.01	Holt-Winters' additive method	23.5660	2.7902
Oomura_PM_2.5_	− 0.557	0.178	0.1515	0.04	ETS(M,A,A)	22.0190	2.4334
Kawadana_PM_2.5_	− 0.467	0.204	0.1516	0.03	ARIMA(1,0,1) with non-zero mean	23.9933	2.4873
Togitsue_PM_2.5_	− 0.581	0.081	0.1360	0.01	Holt-Winters' additive method	21.0916	2.3016
Yukiura_PM_2.5_	− 0.574	0.045	0.1334	0.01	ETS(M,Ad,A)	21.9601	2.2308
Matsuura Shisamachi_PM_2.5_	− 0.605	0.188	0.1602	0.02	ARIMA(2,0,0)(0,0,1)[12] with non-zero mean	18.1827	2.0515
Tsushima_PM_2.5_	− 0.581	0.168	0.1701	0.01	ARIMA(0,1,1)(2,1,0)[12]	12.6324	1.5047
Iki_PM_2.5_	− 0.718	0.005	0.1547	0.01	ARIMA(0,0,0)(2,1,0)[12] with drift	19.8552	2.5151
Goto_PM_2.5_	− 0.429	0.014	0.1470	0.01	Holt-Winters' additive method	18.1000	2.2244
Obama_PM_2.5_	− 0.542	0.064	0.1347	0.02	ARIMA(0,1,1)(1,0,0)[12]	17.3931	1.7036
Kogakura_PM_2.5_	− 1.014	0.001	0.1138	0.01	Holt-Winters' additive method	12.5448	1.7062
Inasa_PM_2.5_	− 0.824	0.003	0.1173	0.01	ARIMA(1,1,1)(1,0,0)[12]	15.9630	1.9276
Muramatsu_PM_2.5_	− 0.865	0.002	0.1227	0.01	ETS(M,Ad,A)	14.2451	1.6485
Higashi Nagasaki_PM_2.5_	− 0.833	0.003	0.1108	0.01	Holt-Winters' additive method	11.9808	1.5653
Fukuishi_Jihai_PM_2.5_	− 1.178	0.002	0.1400	0.01	ETS(M,A,M)	14.8669	1.8090
Yoshii_PM_2.5_	− 0.559	0.003	0.1414	0.01	ETS(M,A,A)	26.5897	2.5581
Daitou_NO_2_	− 0.414	0.003	0.2773	0.01	ETS(A,A,A)	8.6719	0.7435
Isahaya_NO_2_	− 0.218	0.028	0.4277	0.01	Holt-Winters' additive method	34.2538	1.3374
Shimabara_NO_2_	− 0.162	0.032	0.1629	0.01	Holt-Winters' additive method	30.5764	1.4037
Oomura_NO_2_	− 0.262	0.043	0.2977	0.01	ETS(A,A,A)	15.8421	0.8721
Kawadana_NO_2_	− 0.179	0.006	0.3297	0.01	ARIMA(1,0,0)(2,1,1)[12] with drift	8.6144	0.4449
Togitsue_NO_2_	− 0.194	0.007	0.3879	0.01	ARIMA(0,0,0)(0,1,1)[12] with drift	13.2104	0.8717
Yukiura_NO_2_	− 0.016	0.332	0.1968	0.01	ARIMA(0,0,1)(2,1,0)[12]	20.5383	0.4300
Matsuura Shisamachi_NO_2_	− 0.123	0.026	0.2012	0.01	Holt-Winters' additive method	16.8278	0.5062
Tsushima_NO_2_	− 0.032	0.394	0.3543	0.01	Holt-Winters' additive method	10.1772	0.4442
Iki_NO_2_	− 0.035	0.633	0.2060	0.01	Holt-Winters' additive method	43.7718	1.3767
Goto_NO_2_	− 0.250	0.034	0.2290	0.01	Holt-Winters' additive method	26.8487	0.6317
Obama_NO_2_	− 0.085	0.060	0.2679	0.01	ARIMA(1,0,0)(2,1,0)[12]	32.7484	0.6606
Kogakura_NO_2_	− 0.488	0.002	0.2506	0.01	ARIMA(1,0,0)(2,1,0)[12] with drift	21.6239	1.4213
Inasa_NO_2_	− 0.247	0.004	0.2993	0.01	ETS(A,A,A)	14.3113	0.8219
Muramatsu_NO_2_	− 0.145	0.296	0.2812	0.01	ARIMA(0,1,0)(0,1,2)[12]	21.0657	0.9726
Higashi Nagasaki_NO_2_	− 0.501	0.003	0.2849	0.01	ARIMA(0,1,1)(0,1,1)[12]	10.7545	0.6807
Fukuishi_Jihai_NO_2_	− 0.776	0.002	0.1526	0.01	ETS(M,A,M)	7.8342	1.2311
Yoshii_NO_2_	− 0.242	0.002	0.2727	0.01	ETS(A,A,A)	13.5204	0.3613

The results of Holt-Winters and ARIMA forecast analysis are presented in Table 5, which indicated, based on the MAPE and RMSE, that model suitability to forecast PM_2.5_ and NO_2_ varies depending on the location of the monitoring station. For PM_2.5_, Isahaya, Kawadana, Matsuura Shisamachi, Tsushima, Iki, Obama, and Inasa ARIMA gave better results; ETS and Holt-Winters gave better results in the other monitoring stations. In the case of NO_2_, ETS and Holt-Winters gave better results in Daitou, Isahaya, Shimabara, Oomura, Matsuura Shisamachi, Tsushima, Iki, Goto, Inasa, Fukuishi Jihai, and Yoshii with ARIMA providing better results in the other stations. The highest MAPE for Holt-Winters for PM_2.5_ was in Shimabara (23.566), and the lowest was in Higashi Nagasaki (11.98). For ETS, the highest MAPE was in Yoshii (26.59), the lowest was in Muramatsu (14.25), and for ARIMA, the highest MAPE was in Kawadana (23.99), and the lowest was in Tsushima (12.63). For NO_2_, the highest RMSE for Holt-Winters was in Iki (43.77), the lowest was in Tsushima (10.18), and for ETS, the highest was in Oomura (15.84), and the lowest was in Fukuishi Jihai (7.83), and for ARIMA the highest was in Obama (32.75), and the lowest was in Kawadana (8.61).

**Figure 6:**
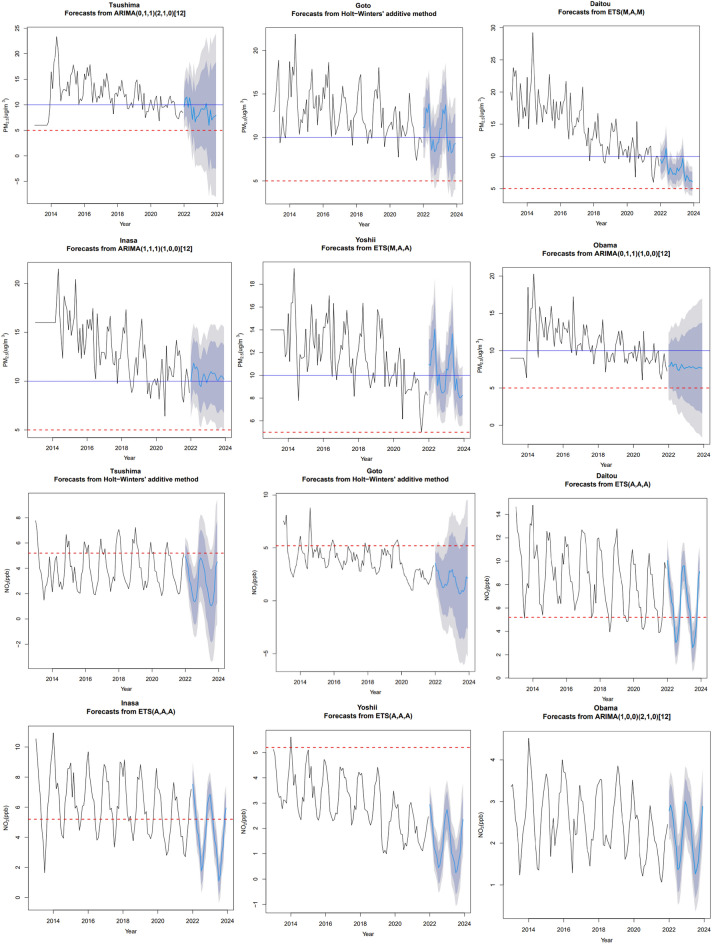
Models and Forecast of PM_2.5_ and NO_2_ for different monitoring stations in Nagasaki Prefecture, Japan. Blue and red lines represent the WHO PM_2.5_ and NO_2_ recommendations for 2005 and 2021, respectively.

Figure 6 shows the forecast results for PM_2.5_ and NO_2_ for six monitoring stations using the best model, ETS, Holt-Winters, or ARIMA (Table 5). PM_2.5_ for Tsushima, Inasa, Yoshii and Obama was forecasted with ARIMA, while Goto and Daitou were forecasted with Holt winters. The forecast of PM_2.5_ produced by ARIMA in Inasa and Obama tends to converge to the mean. In Tsushima, the ARIMA model; in Goto, the Holt-Winters model; and in Daitou and Yoshii, the ETS model was able to replicate the trend and the seasonal components of the data for PM_2.5_. For NO_2_, Tsushima and Goto were forecasted with Holt-Winters, Obama was forecasted with ARIMA, and the other stations were forecasted with ETS. For NO_2_ ETS, Holt–Winters and ARIMA were able to replicate the data's trend and seasonal components. However, the tendency of the data to converge towards the mean was not observed in the case of NO_2_.

For PM_2.5_, in general, the future forecast indicates a negative trend. However, the future concentration of PM_2.5_ will remain above the 2021 WHO recommendations (5 μg/m^3^). Also, in most stations, the future concentrations will stay above or below the 2005 WHO recommendations (10 μg/m^3^), depending on the season, except for Daitou, which shows that the future concentrations will decline below the 2005 recommendations. For NO_2_, the forecast shows a very slight declining trend for the majority of the stations. Compared to the other stations in Yoshii, the decline is more consistent. For NO_2_, all the future forecasts are below the 2005 WHO recommendations (21 ppb). Also, for most stations, the NO_2_ concentration will be below or above the 2021 WHO recommendations (5 ppb), depending on the season. Yoshii and Obama are exceptions, as NO_2_ concentration levels are below the 2021 recommendations.

## Discussion

Acknowledging that air pollution has adverse health effects, even at the lowest observed levels, is crucial for reconsidering current legislation and regulation. Thus, reducing the health impacts caused by the average annual exposure to NO_2_ and PM_2.5_ needs to be prioritized to address known inequities owing to economic activities, socioeconomic conditions, and increased vulnerability of the residential population^8^. Although regulation and legislation in Japan have effectively reduced PM_2.5_ and NO_2_ concentrations over the years, and it is considered one of the industrialized countries with low levels of these pollutants, the results indicate that the average annual concentrations of PM_2.5_ and NO_2_ exceeded the 2021 pollution concertation guidelines of WHO. In particular, PM_2.5_ annual average concentration exceeded the 2005 and 2021 pollution guidelines. The difficulty of regulating and reducing PM_2.5_ concentration in Nagasaki Prefecture is due to the long-range transport of PM_2.5_ from East Asia and Eurasia^28^.

As a result of the long-range transport characteristic of PM_2.5_, its spatial distribution and concentration vary throughout the study period as it is affected by climatic and temporal factors. For instance, Pearson's correlation and the random forest feature selection indicated that the most important factors influencing PM_2.5_ were Spring, maximum instantaneous wind speed, humidity, sunlight time one day, and southern oscillation index. In Spring, PM_2.5_ concentrations are higher than in other seasons (Table S2). This is due to the changes in meteorological conditions, especially wind direction, which affects the long-range transport of PM_2.5_ from East Asia^20^. Maximum instantaneous wind speed was negatively correlated in some stations, showing that horizontal dispersion plays a role in Nagasaki Prefecture. However, maximum wind speed was positively correlated, indicating that PM_2.5_ pollutants are being transported from other areas^29^. This result is further reinforced by Fig. 2, which suggests that from 2014 through 2021, the highest concentration of PM_2.5_ are located in the westernmost part of Nagasaki Prefecture. Several studies have indicated that PM_2.5_ is transported to Nagasaki Prefecture from East Asia; thus, the proximity of the westernmost part of Nagasaki's Prefecture to East Asia, its downwind location, and the change of wind direction in Spring are the main reasons for high PM_2.5_ concentrations detected during the study period. The less affected areas are those located in the easternmost part, which is further away from East Asia. The wide distribution of PM_2.5_ and its spatial variability thought the study period makes it difficult to regulate and identify specific hotspots. Its wide distribution is also a cause for concern as it has health implications for many of the resident population in Nagasaki Prefecture. However, during the study period, as indicated by Table 2, Figs. 2, 3, and 5, there has been a decline in PM_2.5_ concentrations. This decline in PM_2.5_ in Nagasaki Prefecture is related to the decrease in PM_2.5_ concentrations in China and Korea. This reduction can be attributed to the changes in policy, technology, social, environmental, and economic factors in Japan, Korea, and China. For instance, the changes in environmental policies and the tri-national cooperation between these countries have generated positive results in reducing PM_2.5_ (see Section “Policy background” Table 1). Also, the restrictions on social and economic activities imposed due to the COVID-19 pandemic resulted in a notable reduction of PM_2.5_ in 2020 and 2021. Although PM_2.5_ shows a declining trend, better local and regional strategies are needed to reduce PM_2.5_ further as the pollution levels are above the WHO guidelines amidst local and tri-national efforts.

As for NO_2_, the average annual concentration is below the 2005 pollution guidelines. However, the results indicated that the hotspots identified are above the WHO 2021 pollution concentration guidelines. NO_2_ pollution concentrations are also influenced by climatic and temporal factors, as indicated by Pearson's correlation and random forest feature selection analysis. For NO_2_, average wind speed was negatively correlated due to the dilution and dispersion of pollutants. However, maximum wind speed and maximum instantaneous windspeed were positively correlated, which can be attributed to the notion that the NO_2_ plum is buoyant, but at higher wind speeds, the plum is brought down to ground level^30^. Temperature was negatively correlated with NO_2_; temperature is known to promote air convection, leading to pollution dispersion and dilution^31^. Average local pressure and average sea level pressure were positively correlated due to the low atmospheric boundary layer, which accompanies high pressure and prevents air pollutants' vertical dispersion^29^. Sunlight time was negatively correlated to NO_2_; this can be attributed to the photochemical reactions of solar radiation, which reduced NO_2_ concentration. Amidst the influence of climatic factors on NO_2,_ its spatial distribution remained constant throughout the study period with consistent hotspot areas, except for 2020, where the pollution concertation was the lowest and more dispersed with no visible hotspot. From 2013 to 2019 and 2021, hotspots were located in Nagasaki's Prefecture major cities, Nagasaki, Sasebo, Isahaya, and Oomura. Nagasaki and Sasebo are the two largest cities in Nagasaki Prefecture with the highest concentrations of NO_2_ throughout the study period; this is because of the economic activities in the area associated with shipbuilding, power plants, machinery, and heavy industries and also the burning of fossil fuels, especially from the transport sector. The lowest concentrations of NO_2_ were in 2020–2021, as indicated by Table 2 and Fig. 3; this remarkable reduction of NO_2_ can be attributed to the restrictions imposed by the Japanese government on social and economic activities due to the COVID-19 pandemic. The reduction of NO_2_ in 2018–2019 could be due to the decommissioning of the Ainoura Power Station, a crude oil-fired power plant. The more gradual decrease of NO_2_ during the study period, as indicated by the trend and forecast analysis, can be attributed, among other factors, to the stricter vehicle emission regulations implemented^22^ and also the regulation of emissions from stationary sources such as fossil fuel powerplants, electric and industrial boilers.

Pearson's correlation and the random forest feature selection identified major factors influencing PM_2.5_ and NO_2_ and provided a good indication of the complex relationship between the significant climatic and temporal factors and PM_2.5_ and NO_2_ pollutants in Nagasaki Prefecture. However, the results indicate that the correlation and factors of importance that influence PM_2.5_ and NO_2_ vary depending on the monitoring station. These differences observed in terms of correlation, factors of importance, tend, and model performance among the 18 stations can be attributed to the varying unique characteristics of climatic, environmental, social, and economic factors in each location, which affect PM_2.5_ and NO_2_ concentrations. For instance, in the case of Goto_,_ the major predictor of PM_2.5_ is Spring and humidity in Obama. This difference can be attributed to the location of these monitoring stations. Goto is located in the westernmost part of Nagasaki Prefecture, which is the area most affected by the long-range transport of PM_2.5_ from East Asia in Spring, as opposed to Obama, which is located in the easternmost part of Nagasaki Prefecture, which is the least affected by the seasonal changes. Although RF was able to identify the major factors influencing PM_2.5_ and NO_2_, the model's prediction of PM_2.5_ and NO_2_ can be further improved by including not only climatic and temporal factors but emission sources and factors related to human activities such as economic development, transportation, and energy utilization^32^. And in the case of PM_2.5_, including emission sources and human activity factors from China and Korea can improve the model's predictive capabilities. Therefore, even though this study has generated valuable information on the spatiotemporal distribution, tend, influencing factors, and forecast of PM_2.5_ and NO_2_ in Nagasaki Prefecture, additional studies are needed to evaluate further the influence of social, environmental, economic, and technological factors affecting the spatiotemporal distribution and trend of PM_2.5_ and NO_2_ in Nagasaki Prefecture. And also to assess the differences that exist (e.g., trend, influencing factors, etc.) among the monitoring stations.

## Materials and methods

### Study site

**Figure 7:**
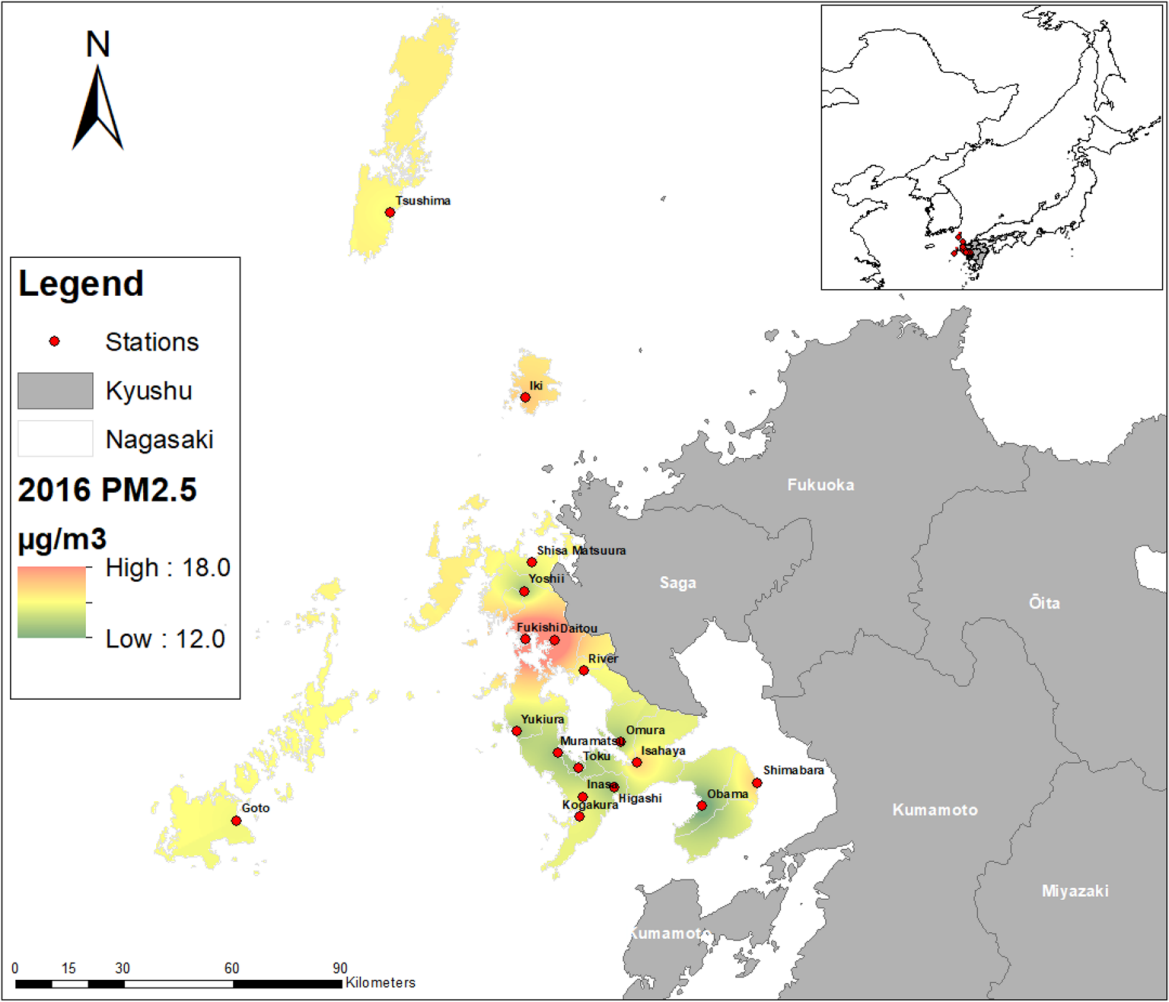
Study site and air pollution monitoring stations in Nagasaki Prefecture, Japan. Created with ArcMap 10.7 (https://www.arcgis.com/index.html).

Nagasaki Prefecture is located on the island of Kyushu (Fig. 7). The prefecture has an area of approximately 4,105 km^2^ with a population of 1,377,187. Nagasaki borders Saga Prefecture on the east and is surrounded by the Tsushima Straits, the Ariake Bay, and the East China Sea. Nagasaki air pollution is relatively low but is influenced by transboundary air pollution from Asia and Eurasia^28,33,34^. Studies conducted in Nagasaki have demonstrated that air pollution has adverse health effects, especially in children^14,16^. Moreover, 8.3 and 29.6% of the population in Nagasaki Prefecture are less than or equal to 10 and more than or equal to 65 years of age, respectively. Therefore, they are considered vulnerable to air pollutants^35^. Although up until March 2012, Nagasaki Prefecture had no PM_2.5_ monitoring stations, the first two stations to record PM_2.5_ concentration were installed in Isahaya and Iki.

### Air pollution datasets

The monitoring station network in Nagasaki Prefecture has increased from four monitoring stations in March 2012 to 18 monitoring stations. The recorded data of these eighteen stations are available from the Nagasaki Prefecture Atmospheric Environment Information (http://www.pref.nagasaki.jp/). PM_2.5_ and NO_2_ data is collected daily at one-hour intervals, from which monthly and annual averages are calculated. Monitoring stations are located at municipal offices, elementary schools, and towers. We selected the 2013–2021 PM_2.5_ and NO_2_ datasets because measurements of these pollutants were collected at each of the 18 monitoring stations for each year of the study period. For this study, we calculated the monthly mean concentration of PM_2.5_ and NO_2_ for each of the 18 monitoring stations. In Japan, NO_2_ concentrations are given in parts per billion (ppb) as opposed to PM_2.5_ measurements, which are given in micrograms per cubic meter (μg/m^3^). Climatic data of Nagasaki from 2013 – 2021 were collected from the Japan Meteorological Agency website (Table 6).

**Table 6:** Parameters for the random forest model.

Type	Parameter	Symbol	Unit (Daily)	Location
Air pollutant	PM_2.5_	PM_2.5_	μg/m^3^	18 Point (Fig. 7)
	NO_2_	NO_2_	ppb	18 Point (Fig. 7)
Climatic factors	Average local pressure	LP	hPa	Data from
	Average sea level pressure	SP	hPa	Japan Meteorological
	Daily Precipitation	Rain	mm	Agency in Nagasaki^1)^
	Maximum 1-h Precipitation	Rain1h	mm	
	Maximum 10 min Precipitation	Rain10m	mm	
	Average temperature	Temp	℃	
	Maximum temperature	Max.T	℃	
	Minimum temperature	Min.T	℃	
	Average humidity	Humid	%	
	Minimum humidity	Min.H	%	
	Average wind speed	WindS	m/s	
	Maximum wind speed	MWS	m/s	
	Maximum instantaneous wind speed	MIWS	m/s	
	Sunlight time	SUN	h	
Temporal factors	Wind direction	WindD	8 Directions	Nagasaki^1)^
	Seasons	Season	4 Seasons	Japan
	Japan Day	Day	Holiday or not	Japan
	Arctic Oscillation index	AOI	6 Rank	NOAA CPC^2)^
	Southern Oscillation Index	SOI	Positive or negative	Queensland^3)^

^a^www.jma.go.jp.

^b^www.cpc.ncep.noaa.gov/products/precip/CWlink/daily_ao_index/ao.shtml.

^c^www.longpaddock.qld.gov.au/soi.

*Time lag data (1–7 days past each day) was created for the climatic variables.

### Data processing and ordinary kriging

For the 18 stations, we calculated the annual average for PM_2.5_ and NO_2_ from the daily data collected in each monitoring station from 2013 to 2021. This resulted in nine datasets for PM_2.5_ and nine for NO_2_, which were used to implement ordinary kriging to predict the spatiotemporal distribution. The dataset for each year was divided into four seasons, Spring: March to May, Summer: June to August, Autumn: September to November, and Winter: December to February, and summary statistics were calculated (Table S2).

### Ordinary kriging

Ordinary kriging (OK) interpolation is suitable for PM_2.5_ and NO_2_ concentration mapping as it is a commonly used geostatistical estimator in air pollution interpolation and is often referred to as the unbiased estimator^36^. Ordinary kriging models the unsampled value z*(${x}_{0}$) as a combination of neighboring observations n, Eq. (1),^37^:

1 $$z^{*} \left( {x_{0} } \right) = \mathop \sum \limits_{i = 1}^{n} {\uplambda }_{i} Z(x_{i} )$$

where z*(x_0_) estimate value at x_0_, Z(x_i_) measure value at x_i_ and λ_i_ weight is assigned for the residual of Z(x_i_).

#### Semivariogram

We derived the experimental semi-variogram for the 18 datasets to determine the spatial autocorrelation and the spatial structure of data points. The semi-variograms are expressed as a function of the distance between data points and explain the measured points' spatial relationship, Eq. (2),^38^.

2 $${\upgamma }\left( {\text{h}} \right) = \frac{1}{2N\left( h \right)} \mathop \sum \limits_{i = 1}^{N} \left[ {Z\left( {x_{i} + h} \right) - Z\left( {x_{i} } \right)} \right]$$

where $\upgamma$(h) quantity function of increment h, N(h) numbers of pairs separated by the vector h, Z(x_i_) is the sampled values at location x_i_ and Z(x_i_ + h) sampled measurements at location X_i_ + h.

In this study, we fitted the experimental semi-variogram to two theoretical semi-variogram models: exponential and spherical, two of the most commonly used models^39^. The parameters determined were: range (a) the distance up until which the regionalized variable is auto-correlated, partial sill (c) which is the spatially structured part of the residuals, and the nugget (c_0_) the non-spatial variability^40^. The spherical and exponential models are defined by Eqs. (3) and (4) ^41^.

3 $$\begin{gathered} Exponential\;Model \hfill \\ \gamma \left( h \right) = c_{0} + c\left[ {1 - \exp \left( { - \frac{3h}{a}} \right)} \right] \hfill \\ \end{gathered}$$

4 $$\begin{gathered} Spherical\;Model{ } \hfill \\ \left\{ \begin{gathered} \gamma \left( h \right) = c_{0} + c\left[ {1.5\left( \frac{h}{a} \right) - 0.5\left( \frac{h}{a} \right)^{3} } \right],\quad \;h \le a \hfill \\ \gamma \left( h \right) = c_{0} + c,\quad \quad \quad \quad \quad \quad \quad \quad \quad \quad \quad \quad h > a \hfill \\ \end{gathered} \right. \hfill \\ \end{gathered}$$

#### Cross validation

The model's prediction ability of the unsampled PM_2.5_ and NO_2_ locations was conducted using cross-validations to calculate the mean error (ME), standard mean error (SME), root mean square error (RMSE), root mean square standard error (RMSSE) and average standard error (ASE). We analyzed the cross-validation results for both spherical and exponential models; the model with better results was selected for interpolating PM_2.5_ and NO_2_ (Table S1). The RMSE and RMSSE are defined by Eqs. (5) and (6), respectively^42^.

5 $${\text{RMSE}} = { }\sqrt {\frac{1}{N}\mathop \sum \limits_{i = 1}^{N} \left[ {Z\left( {x_{i} } \right) - Z^{*} \left( {x_{i} } \right)} \right]^{2} }$$

6 $${\text{RMSSE}} = { }\sqrt {\frac{1}{N} \mathop \sum \limits_{i = 1}^{N} \left[ {Z_{1} \left( {x_{i} } \right) - Z_{2} \left( {x_{i} } \right)} \right]^{2} }$$

where N number of validation points, Z(x_i_) measured value and Z*(x_i_) standard values being Z_1_(x_i_) and Z_2_(x_i_).

A RMSE closer to 0 and a RMSSE closer to 1 depict that the parameters and fitting model are excellent and the kriging estimators are robust.

### Pearson’s correlation and random forest

Pearson's correlation analysis was conducted for each of the 18 monitoring stations to produce a heatmap depicting the correlation between major climatic and temporal factors and PM_2.5_ and NO_2_ pollutants (Table 6). We then used random forest (RF) to identify the most important climatic and temporal factors influencing PM_2.5_ and NO_2_ in each of the 18 stations^43^. The factors identified were then used to construct the RF models for each of the 18 stations to make PM_2.5_ and NO_2_ predictions. For each of the 18 stations, the random forest models were trained with 80% and validated with 20% of the respective monitoring station data. The RF model was then evaluated using the root mean square error (R^2^) and the mean, standard error (MSE). Random forest modeling is a type of ensemble learning method used for classification and regression analysis. It is well known to have advantages in terms of accuracy, robustness, and computational efficiency compared to other models^44^. The RF model was constructed using the open-source machine learning library scikit-learn^*2^ on Python. Next, the categorical factors were converted into dummy or indicator factors using the Python Pandas method (get dummies) (Tables 6 and 7). Furthermore, 7-day time lag data was added to the climatic factors to confirm the influence of past dependent factors. Finally, hyperparameters were determined with the ranges and steps indicated in (Table 8) using the grid-search technique for optimal model construction.

**Table 7:** Categorical rank from the continuous data of AOI.

Rank	AOI
−AAA	< − 2
−AA	− 2 to − 1
−A	− 1–0
+A	0–1
+AA	1–2
+AAA	2 <

**Table 8:** Hyperparameters of the random forest grid search.

Parameter	Range	Step
Max_depth	3–7	1
Min_sample_leaf	2–30	2
n_estimators	10–150	10
Mas_features	Auto	

### Trend and forecast analysis

R statistical software was used to conduct the trend and forecast analysis (R Core Team, 2022). For the trend analysis, monthly mean concentrations for PM_2.5_ and NO_2_ were utilized. The csmk.test function was used to conduct the Mann–Kendall test for trend detection. Equation (7) gives Mann–Kendall Statistics *S*, Variance V(S), and standardized test statistics *Z*^45,46^.

7 $$S = \mathop \sum \limits_{i = 1}^{n - 1} \mathop \sum \limits_{j = i + 1}^{n} sgn\left( {x_{j } - x_{i} } \right),$$

$$sgn\left( {x_{j } - x_{i} } \right) = \left\{ {\begin{array}{*{20}c} { + 1, > \left( {x_{j } - x_{i} } \right)} \\ {0, = \left( {x_{j } - x_{i} } \right)} \\ { - 1, < \left( {x_{j } - x_{i} } \right)} \\ \end{array} } \right.$$

$$V\left( S \right) = \frac{1}{18}\left[ {n\left( {n - 1} \right)\left( {2n + 5} \right) - \mathop \sum \limits_{p = 1}^{q} t_{p} (t_{p} - 1)\left( {2t_{p} + 5} \right)} \right],$$

$$Z = \left\{ \begin{gathered} \frac{S - 1}{{\surd var\left( s \right)}}\quad if\;S > 0 \hfill \\ 0\quad \quad \quad \quad \;if\;S = 0 \hfill \\ \frac{S + 1}{{\surd var\left( S \right)}}\;\;if\;S < 0 \hfill \\ \end{gathered} \right.$$

where x_j_ and x_i_ time series and n number of data points in the time series. Where t_p_ number of ties up to sample p. A positive Z value signifies a rising trend, a negative Z signifies a descending trend for the data period.

The sens.slope function was used to calculate the Sen's slope which indicated the magnitude of the trend. Equation (8) gives the slope for all data pairs and Eq. (9) the median of the n values of T_i_, Sen's slope estimator (Q_i_)^47^.

8 $$T_{i} = \frac{{x_{j} - x_{k} }}{j - k}$$

where Ti slope and x_j_ and x_k_ data values at time j and k.

9 $$Q_{i} = \left\{ \begin{gathered} T_{{\frac{n + 1}{2}}} ,\quad \quad \quad \quad \quad \;n\;is\;odd \hfill \\ \frac{1}{2}\left( {T_{\frac{n}{2}} + T_{{\frac{n + 2}{2}}} } \right),\;n\;is\;even \hfill \\ \end{gathered} \right.$$

A positive *Q*_*i*_ signifies a rising trend; a negative *Q*_*i*_ signifies a declining trend over time.

Both Mann–Kendall and Sen’s slope consider the seasonality of the data. The trend package in R was used to do the correlated seasonal Man-Kendall test and the seasonal Sen's slope tests^48^. Both functions do not operate on missing data; therefore, the tsclean function in the forecast package was used^49,50^. To obtain the trend of PM_2.5_ and NO_2_, we decompose the time series data into a trend, seasonal and irregular components by using the stl (seasonal decomposition of time series by LOESS) function developed by William Cleveland^51,52^. The stl function from the stats package was used to fit the loess to the data and the tsclean function in the forecast package was used to identify and replace outliers and missing values before applying the stl function. Then the stl function from the stats package was used to fit the loess to the data. The mean absolute percentage error (MAPE) and root mean square error was computed to determine if the component after the LOESS decomposition had satisfactorily captured the PM_2.5_ and NO_2_ data information. The goodness of fit of the trend line was determined by checking the residuals; this was done by using the checkresiduals function from the forecast package.

Both exponential smoothing and ARIMA models were evaluated for the forecast analysis. These methods have been used to perform air pollution forecast analysis and, in some cases, have performed better than deep learning methods. First, the Augmented Dickey-Fuller test *(*ADF Test) was performed to ensure the stationarity of the time-series data^53^. Once stationarity was confirmed, the two models were trained and tested with the 2013–2019 and the 2020–2021 pollutants datasets, respectively. Next, validation was performed using the test set whereby the mean absolute percentage error (MAPE) and root mean square error were computed to determine if the EST and ARIMA had satisfactorily captured the information of the PM_2.5_ and NO_2_ data. The models with the lowest AIC were then used to do the forecasting of both PM_2.5_ and NO_2_.

#### Exponential smoothing (ES) forecasting methods and models

Brown, Winter and Holt introduced the exponential smoothing^54^. Gardner^55^ extensively reviews the various ES methods. The exponential smoothing forecasting formulation consists of the forecast method and the statistical model. The forecast method uses an algorithm to produce a point forecast which is a prediction of a single value whereas the forecast statistical model is a process which generated an entire probability distribution with several values which when averaged generates a point forecast and provides prediction intervals with a level of confidence^54^.

The exponential smoothing forecasting method is based on the idea that the forecast produced are weighted averages of past observations, with the weight associated to each observation exponentially decreasing as the observation gets older^54,55^. Model formulations are of component (recursive) form and error correction form^54^. The error correction form is derived from the rearrangement of the equation in the component form. This error correction form uses the state space approach to exponential smoothing method since it consists of a measurement (observed) equation and a state (transition) equation. These two equations with their error distribution constitute a specified statistical model know as state space model. Since all observations and state variables uses the same error process it is called "single source of error" (SSOE) or "innovation" and more specifically known as "innovation state space model. The single source of error (SSOE) was formulated by Snyder^56^.

Pegels provided classification of the trend and the seasonal patterns depending on whether they are additive (linear) or multiplicative (nonlinear)^56^. The family of exponential smoothing forecasting methods can be systematically described as a combination of level, trend, and seasonality^54,58,59^. Each one can be of either an additive character or multiplicative character. The trend component can be classified as having no trend, additive trend, additive damped trend, multiplicative trend, and multiplicative damped trend^55,57–59^. The simplest classification is the single exponential smoothing (SES) method, which considers only the constant level model and uses data with no trend or seasonality. This method consists of a forecast and smoothing equations for the level. The Holt linear trend method, also known as Double Exponential Smoothing (DES), consist of a forecast equation, and two smoothing equations: a level equation and a trend equation.

The Holt-Winters seasonal method, also known as the triple exponential smoothing (TES), consist of a forecast equation and three smoothing equations: a level equation, trend equation, and seasonality equation. The family classification of exponential smoothing generates a combination of 15 exponential smoothing methods with different components^58,59^. Rearranging the terms in the different components for each of the 15 exponential smoothing methods (i.e., level component, trend component, and seasonal component), generate an error correction form model for each of the 15 methods with each having an additive or a multiplicative error model thus producing a total of 30 error models. These error-correction form models, also known as "innovative" state space models, are labeled as ETS ( ; ; ), representing Error, Trend, and Seasonal. The forecast equation is the measurement equation, and the smoothing equations becomes the state equation, with both having the same source of error^54^.

Of the 15 exponential smoothing methods, six were considered here. These are the Holts linear trend method, Holt linear damped trend, Holt-Winters additive, Holt-Winters additive damped, Holt-Winters multiplicative, and Holt-Winters multiplicative damped component. These six methods are converted to their error correction components form with their respective additive and multiplicative error correction model, yielding 12 error correction models. These 12 error correction models were used for model selection (Table S3).

The innovative state space model forecasting for each univariate time series was generated using the ets() function in the forecast package in R^49,58^. Two procedures using ets() function for model selection were used: the automatic selection and the manual selection of a model. The automatic selection of the ETS models provides options for which models to be evaluated and selects the most appropriate model given the data. The model option used was model = "ZAZ" where the first Z represents either additive or multiplicative error, the second Z represents automatic selection in which the choices are no seasonality, additive seasonality, or multiplicative seasonality, and the A represents an additive trend. Based on this "ZAZ" option, 12 models were evaluated from the 30 error models available. Selection of the best-fitted model from the 12 models was based on the minimization of the corrected Akaike Information Criterion (AIC), which avoids over-fitting by considering both goodnesses of fit and model complexity.

The manual model selection procedure used was selecting the hw() function from the forecast package in R. This function selects the Holt-Winters additive model, which corresponds to the ETS(A;A;A) in the ets () function which stands for additive error, additive trend, and additive seasonality^55^. The Ljung–Box Q test was used for residual diagnostics to determine whether the residuals were white-noise sequences. The Box.test was used from the stata package in R.

#### The ARIMA models

Slutsky, Walker, Yaglom, and Yule first articulated autoregressive (AR) and moving average (MA) models. Box & Jenkins integrated the existing knowledge formulating ARIMA, known as the Box-Jenkins approach^60^. An autoregressive model (AR) assumes the forecasted value is a linear combination of the past values of the variable, and moving average models (MA) assumes a linear combination of past forecasting errors. Combining these two models, AR and MA, produces an ARMA model. If the time series is non-stationary then the series are differenced to create stationarity before modeling, then the I is introduced in the ARMA. The I in an ARIMA model represents the integration parameter produced by differencing. The non-seasonal ARIMA models have parameters p, d, and q. The p represents the lag order of the autoregression, the d is the order of the differencing, and the q is the order of the moving average for the non-seasonal part. The seasonal ARIMA, also known as SARIMA, incorporates an additional set of terms, like the ARIMA models, that considers the seasonal effects. The seasonal parameters incorporated are P, D, Q, and m. The P, D, and Q represent the lag order of the autoregression, the order of the differencing, and the order of the moving average for the seasonal part, and the m represents the number of periods in each season. Box et al. and Chatfield^61,62^ expressed the AR(p), MA(p), and ARMA (p, q), mixed seasonal ARMA(p,q)(P,Q)_m_, ARIMA(p,d,q), and mixed seasonal ARIMA -SARIMA(p,d,q)(P,D,Q)_m_ models.

ARIMA forecast was done using the automatic ARIMA algorithm for model selection using the auto.arima() function from the forecast package in the R program^49,50^. Two procedures were used in the automatic selection. The first method used the default settings (restricted models), and the second was full model selection. Sometimes running the full model selection will produce a different optimal model^54^.

## Supplementary Information

Supplementary Information.

## Data Availability

The data supporting this research's findings is provided in the supplementary materials. The raw data used for the study is also available from the corresponding author upon reasonable request.
