# Supplementary material for: Spatiotemporal distribution, trend, forecast, and influencing factors of transboundary and local air pollutants in Nagasaki Prefecture, Japan
Source: Sci Rep. 2023 Jan 16;13:851. doi: 10.1038/s41598-023-27936-2 (PMC9842204; doi:10.1038/s41598-023-27936-2)

**Spatiotemporal Distribution, Trend, Forecast, and Influencing Factors of Transboundary and Local Air Pollutants in Nagasaki Prefecture, Japan**

**Abstract**

The study of PM_2.5_ and NO_2_ has been emphasized in recent years due to their adverse effects on public health. To better understand these pollutants, many studies have researched the spatiotemporal distribution, trend, forecast, or influencing factors of these pollutants. However, rarely studies have combined these to generate a more holistic understanding that can be used to assess air pollution and implement more effective strategies. In this study, we analyze the spatiotemporal distribution, trend, forecast, and factors influencing PM_2.5_ and NO_2_ in Nagasaki Prefecture by using Ordinary kriging, Pearson's correlation, Random forest, Mann-Kendall, Auto-regressive integrated moving average and Error Trend and Seasonal models. The results indicated that PM_2.5,_ due to its long-range transport properties, has a more substantial spatiotemporal variation and affects larger areas in comparison to NO_2_, which is a local pollutant. Despite tri-national efforts, local regulations and legislation have been effective in reducing NO_2_ concentration but less effective in reducing PM_2.5_. This multi-method approach provides a holistic understanding of PM_2.5_ and NO_2_ pollution in Nagasaki prefecture, which can aid in implementing more effective pollution management strategies. It can also be implemented in other regions where studies have only focused on one of the aspects of air pollution and where a holistic understanding of air pollution is lacking.

**Supplementary Material**

**Tables:**

Table S1. PM_2.5_ and NO_2_ spherical and exponential model validation


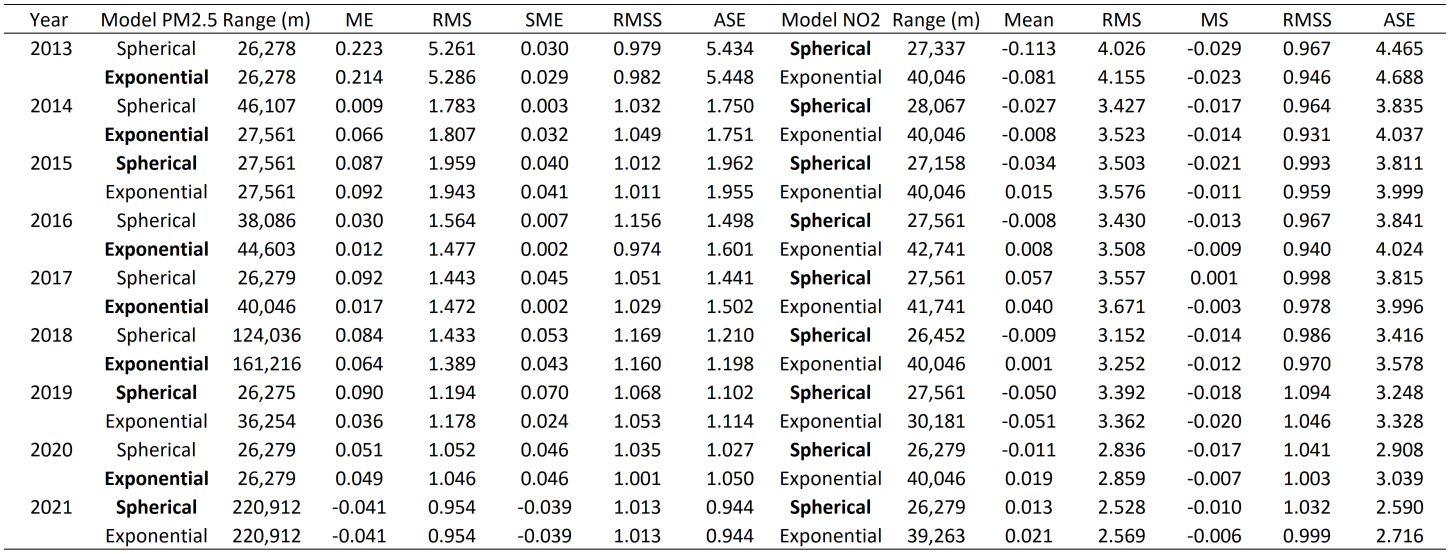
*In bold model used for interpolation


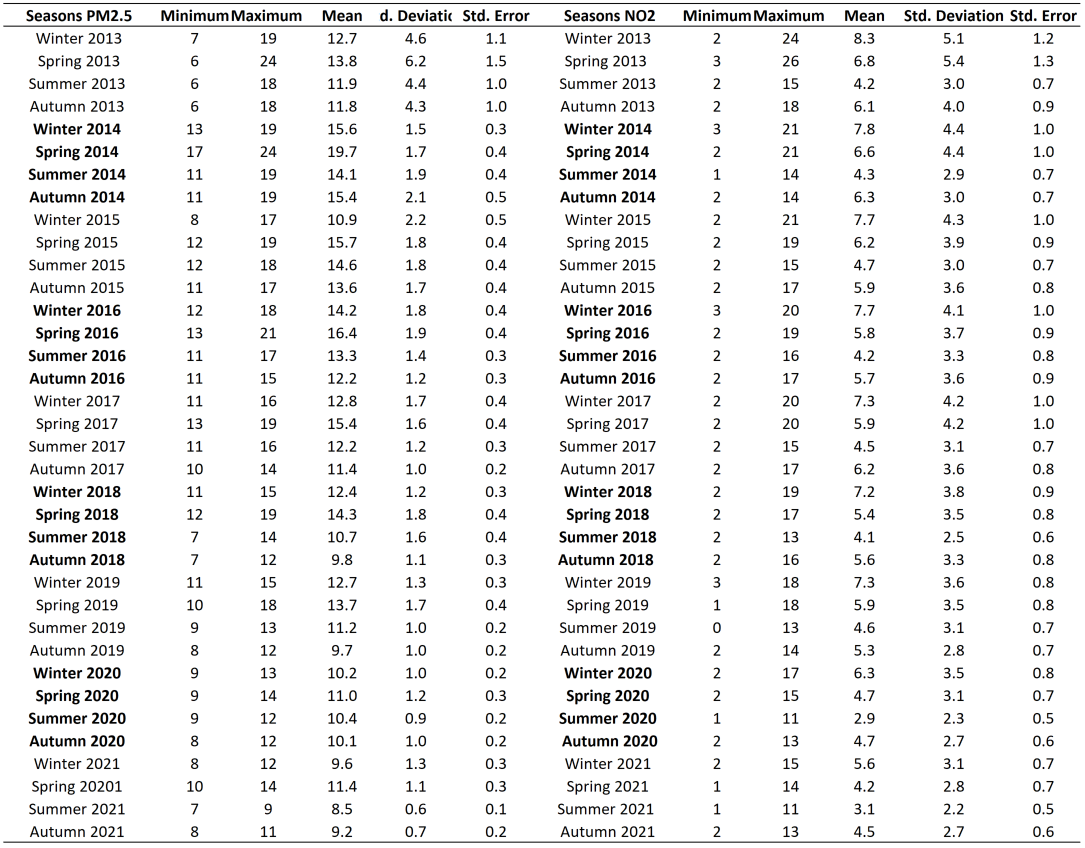
Table S2. Seasonal descriptive statistics for PM_2.5_ and NO_2_ concentrations

Table S3. State space model equation used in the ETS ( ; ; ) modelling framework.

| Error | Trend |  | Seasonal |  |
| --- | --- | --- | --- | --- |
| model |  | N | A | M |
|  |  | *yt* = *lt*−1 + *bt*−1 + *εt* | *yt* = *lt*−1 + *bt*−1 + *st*−*m* + *εt* | *yt* = (*lt*−1 + *bt*−1)*st*−*m* + *εt* |
|  | A | *lt* = *lt*−1 + *bt*−1 + *αεt* | *lt* = *lt*−1 + *bt*−1 + *αεt* | *lt* = *lt*−1 + *bt*−1 + *αεt/st*−*m* |
|  |  | *bt* = *bt*−1 + *βεt* | *bt* = *bt*−1 + *βεt* | *bt* = *bt*−1 + *βεt/st*−*m* |
| A |  |  | *st* = *st*−*m* + *γεt* | *st* = *st*−*m* + *γεt/*(l *t*−1 + *bt*−1) |
|  |  | *yt* = *lt*−1 + *φbt*−1 + *εt* | *yt* = *lt*−1 + *φbt*−1 + *st*−*m* + *εt* | *yt* = (*lt*−1 + *φbt*−1)*st*−*m* + *εt* |
|  | Ad | *lt* = *lt*−1 + *φbt*−1 + *αεt* | *lt* = *lt*−1 + *φbt*−1 + *αεt* | *lt* = *lt*−1 + *φbt*−1 + *αεt/st*−*m* |
|  |  | *bt* = *φbt*−1 + *βεt* | *bt* = *φbt*−1 + *βεt* | *bt* = *φbt*−1 + *βεt/st*−*m* |
|  |  |  | *st* = *st*−*m* + *γεt* | *st* = *st*−*m* + *γεt/*(l *t*−1 + *φbt*−1) |
|  |  | *yt* = (*lt*−1 + *bt*−1)(1 + *εt* ) | *yt* = (*lt*−1 + *bt*−1 + *st*−*m*)(1 + *εt* ) | *yt* = (*lt*−1 + *bt*−1)*st*−*m*(1 + *εt* ) |
|  | A | *lt* = (l*t*−1 + *bt*−1)(1 + *αεt* ) | *lt* = *lt*−1 + *bt*−1 + *α*(*lt*−1 + *bt*−1 + *st*−*m*)*εt* | *lt* = (*lt*−1 + *bt*−1)(1 + *αεt* ) |
|  |  | *bt* = *bt*−1 + *β*( *lt*−1 + *bt*−1)*εt* | *bt* = *bt*−1 + *β*(*lt*−1 + *bt*−1 + *st*−*m*)*εt* | *bt* = *bt*−1 + *β*(*lt*−1 + *bt*−1)*εt* |
| M |  |  | *st* = *st*−*m* + *γ*(*lt*−1 + *bt*−1 + *st*−*m*)*εt* | *st* = *st*−*m*(1 + *γεt* ) |
|  |  | *yt* = (*lt*−1 + *φbt*−1)(1 + *εt* ) | *yt* = (*lt*−1 + *φbt*−1 + *st*−*m*)(1 + *εt* ) | *yt* = (*lt*−1 + *φbt*−1)*st*−*m*(1 + *εt* ) |
|  | Ad | *lt* = (*lt*−1 + *φbt*−1)(1 + *αεt* ) | *lt* =*lt*−1 + *φbt*−1 + *α*(*lt*−1 + *φbt*−1 + *st*−*m*)*εt* | *lt* = (*lt*−1 + *φbt*−1)(1 + *αεt* ) |
|  |  | *bt* = *φbt*−1 + *β*(*lt*−1 + *φbt*−1)*εt* | *bt* = *φbt*−1 + *β*(*lt*−1 + *φbt*−1 + *st*−*m*)*εt* | *bt* = *φbt*−1 + *β*(*lt*−1 + *φbt*−1)*εt* |
|  |  |  | *st* = *st*−*m* + *γ*(*lt*−1 + *φbt*−1 + *st*−*m*)*εt* | *st* = *st*−*m*(1 + *γεt* ) |

* N=none, A= Additive, M=Multiplicative, A_d_= Additive damped. The term yt stands for the observational time series; the terms l_t_, b_t_, and s_t_ stands for level, trend and seasonal components; *α, β, γ* represent smoothing parameters; *φ* represents a damping parameter; m represents number of seasons in a year; and εt denotes the residuals of the errors at time t (adapted from Hyndman 2018)

**Figures:**


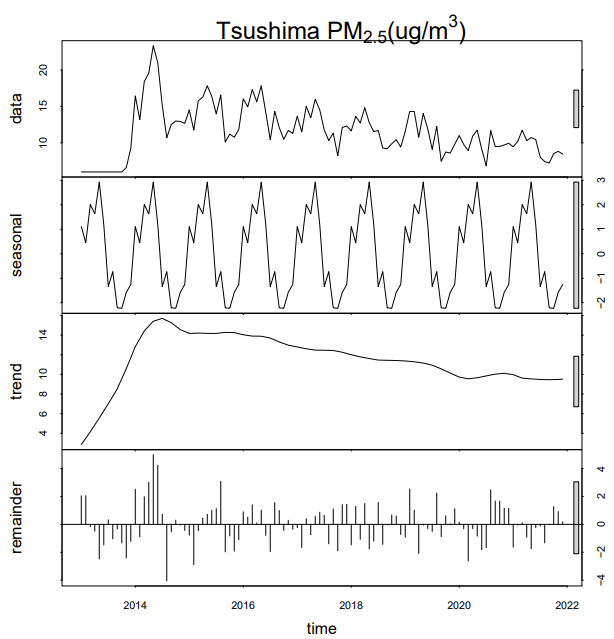

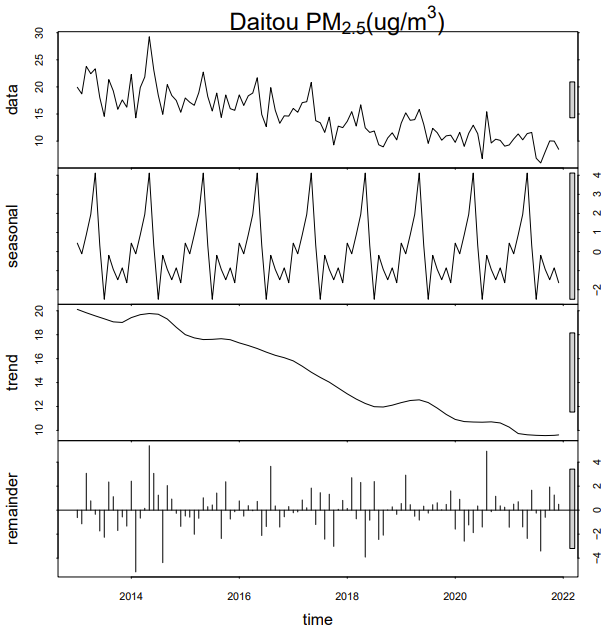

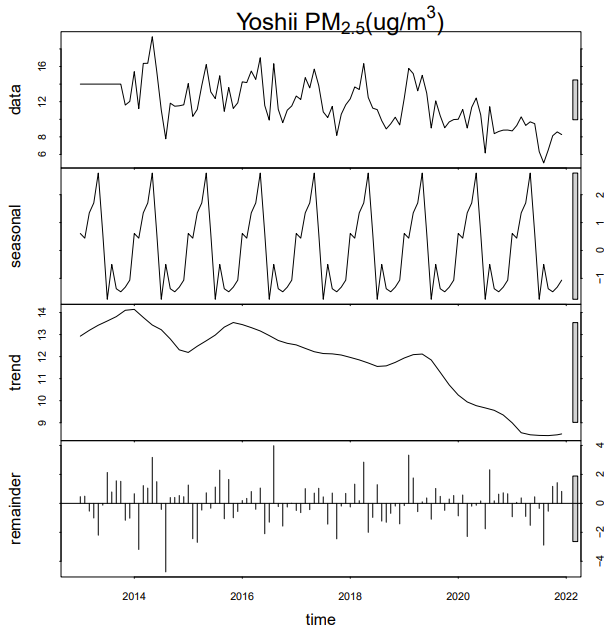

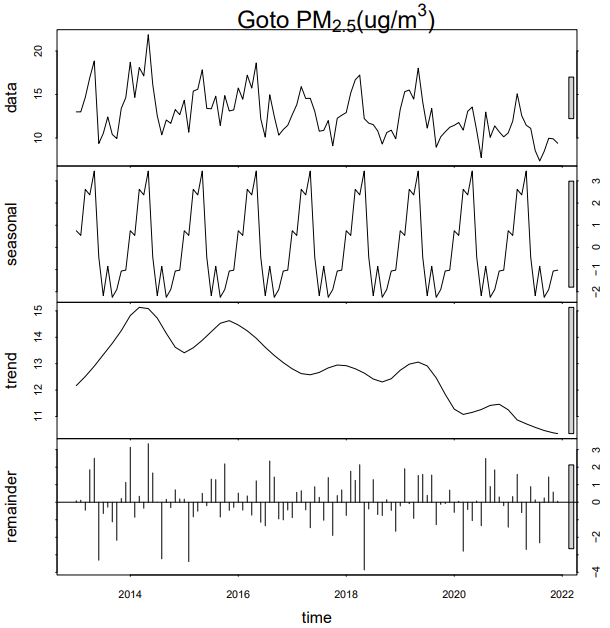

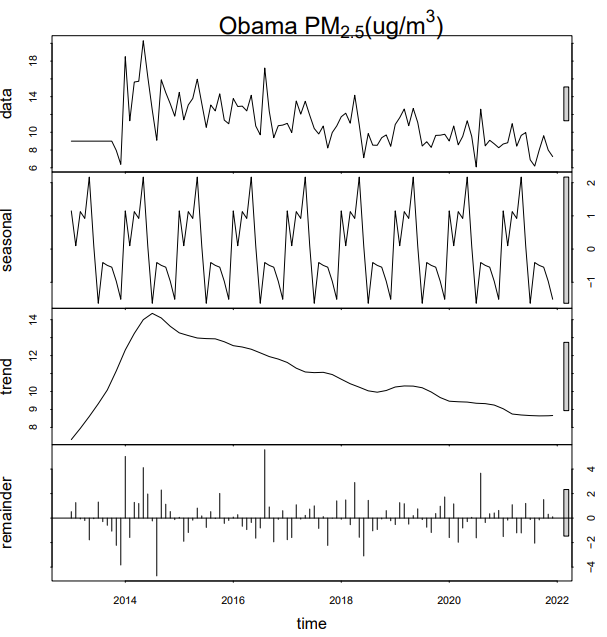

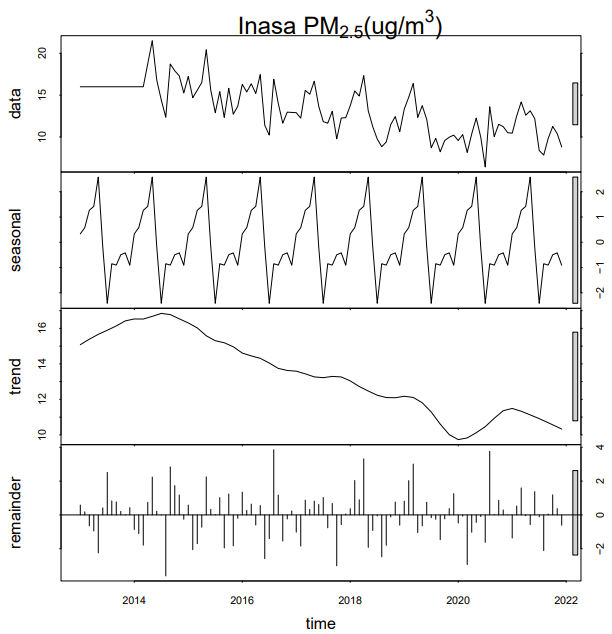


Figure S1. PM_2.5_ data decomposition of monitoring stations in Nagasaki Prefecture, Japan

Figure S2. NO_2_ data decomposition of monitoring stations in Nagasaki Prefecture, Japan


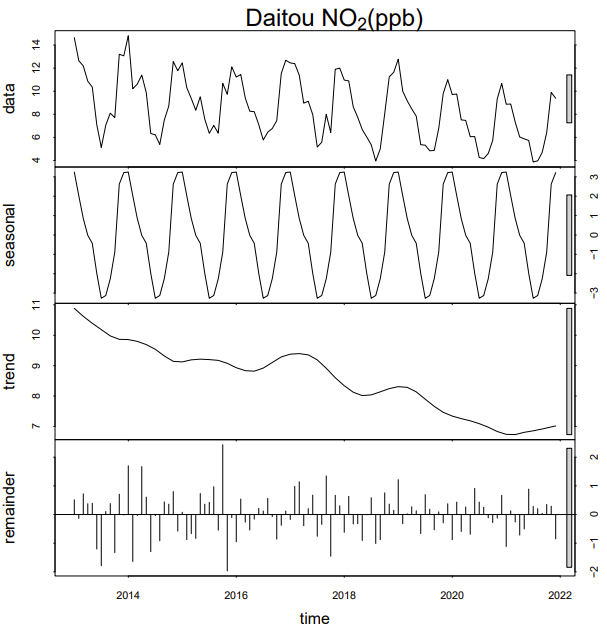

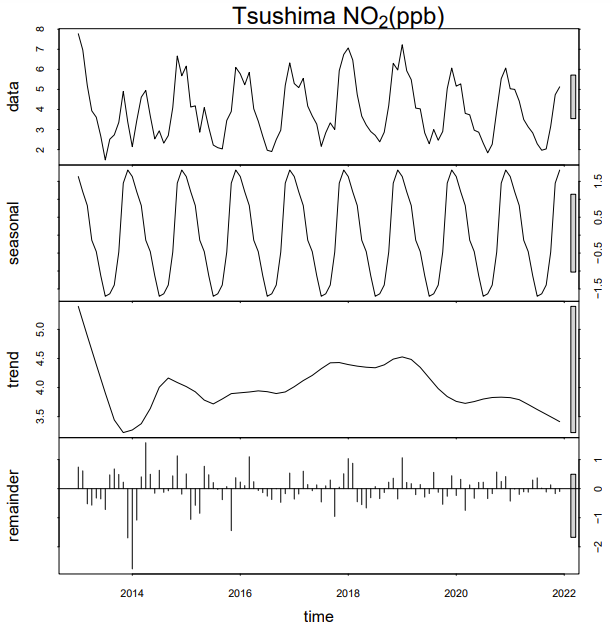

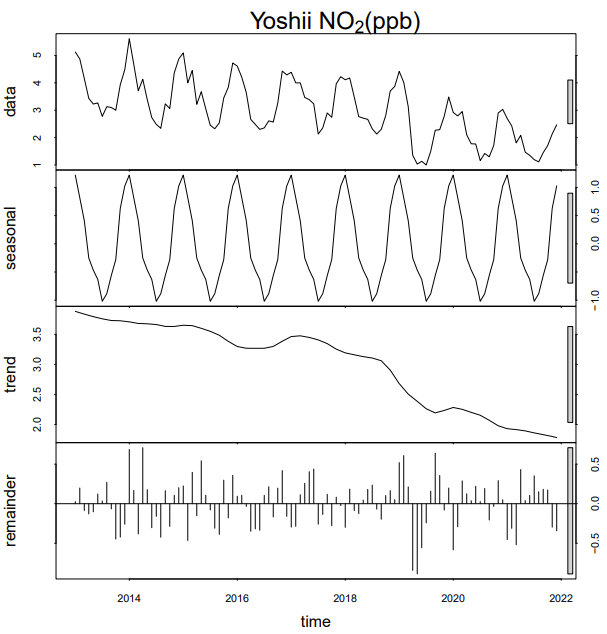

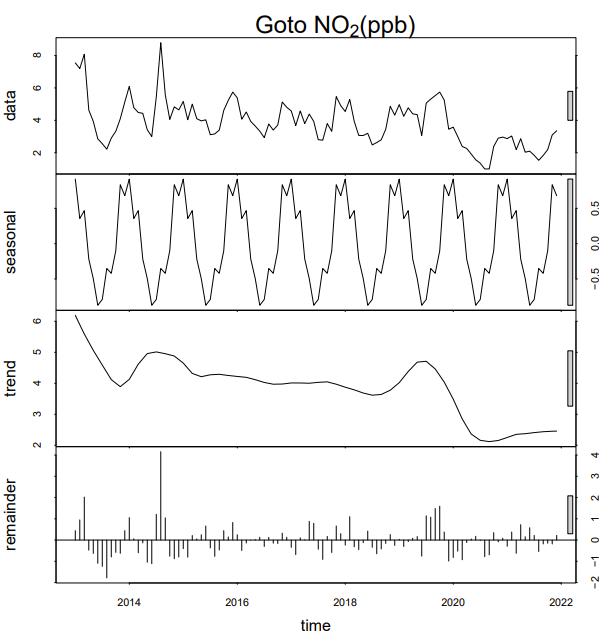

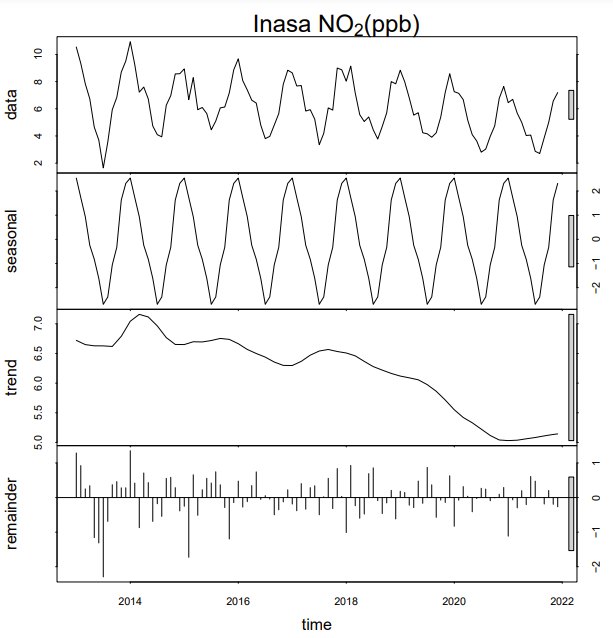

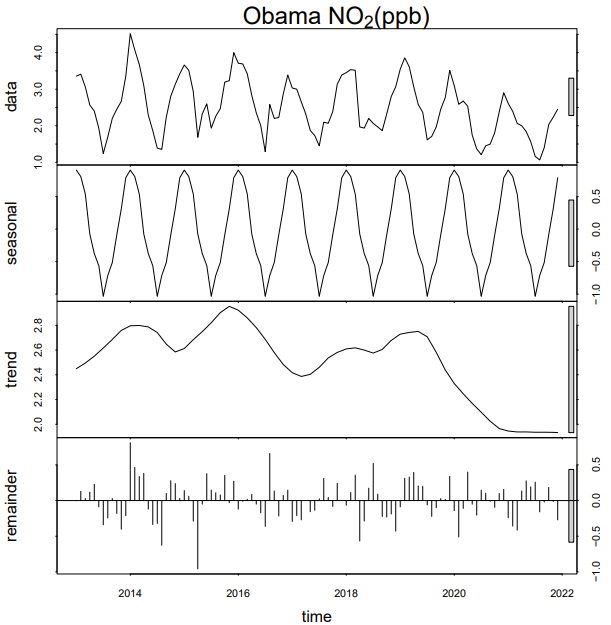

Supplement: Supplementary file 1 — Supplementary Information. [file 41598_2023_27936_MOESM1_ESM.docx]
